# Changes in trunk posture and muscle responses in standing during pregnancy and postpartum

**DOI:** 10.1371/journal.pone.0194853

**Published:** 2018-03-27

**Authors:** Gemma Biviá-Roig, Juan Francisco Lisón, Daniel Sánchez-Zuriaga

**Affiliations:** 1 Department of Physiotherapy, Faculty of Health Sciences, University CEU-Cardenal Herrera, Valencia, Spain; 2 Department of Medicine, Faculty of Health Sciences, University CEU-Cardenal Herrera, Valencia, Spain; 3 Department of Anatomy and Human Embryology, Faculty of Medicine, Universitat de València, Valencia, Spain; West Virginia University, UNITED STATES

## Abstract

The aim of this study was to analyze the position of the lumbopelvic region and the muscle activation of erector spinae and biceps femoris muscles in a group of pregnant women in the third trimester. The hypothesis was that pregnancy-related biomechanical and morphological changes modify the position of the lumbopelvic region and the activation of extensor muscles. The position of the lumbar spine and pelvis in the sagittal plane, and the EMG activity of the erector spinae and biceps femoris muscles, were recorded during standing in 34 nulliparous and 34 pregnant women in the third trimester, and also two months after birth in the group of pregnant women. No significant differences in the position of the lumbar spine or pelvis between the group of pregnant women and nulliparous or postpartum were observed. A significant increase was observed in the EMG activity of the erector spinae (4.6% vs 2.4% and 2.1% in the nulliparous group and postpartum respectively) and the biceps femoris (3.4% vs 1.2% and 1.4%) in pregnant women compared to the other two groups (p <0.01). We conclude that pregnant women in the third trimester show no alterations in lumbopelvic position compared to nulliparous and postpartum women. However, there is an increase of the EMG activity of the trunk extensors. These results indicate that the extensor muscles of the trunk show, in static positions, adaptive responses to the increase of anterior loads during pregnancy.

## Introduction

One of the most frequent complications of pregnancy is low back pain, with a 50–70% prevalence [1–3]. Its incidence is higher in the third trimester of pregnancy, when the most important biomechanical and morphological changes take place. From the second trimester, abdominal morphology is altered by the increased size of the uterus and the weight of the foetus, with a 30% increase in abdominal mass [4]. The increased size of the abdomen has been linked to a decreased static stability [5] and adaptive changes in spinal curvatures, which would compensate the anterior displacement of the centre of gravity, to ensure postural balance. Postural alterations most frequently mentioned in the literature are increased lumbar curvature, pelvic anteversion [6–8], increased thoracic curvature [8], increased cervical curvature, protraction of the shoulder girdle, hyperextended knees [9], and extension of the ankles [10].

The development of back pain has been related to spinal changes, especially an increase in lumbar curvature, which alters the distribution of loads, causing increased tensions in lumbar structures [11,12]. However, changes caused by pregnancy in spinal curvatures in healthy women are not well known, although they may be related to the high occurrence of low back pain in this population. Data from previous studies are controversial: several researchers agree that pregnancy increases lumbar curvature [6–8], whereas others [11,13–16] have not observed such changes. Findings range from an increased lumbar curvature and pelvic anteversion [6] to slight postural adjustments attributed to individual adaptations of each woman [14] or a tendency towards lumbar kyphosis and posterior inclination of the sacrum [15]. The use of different measurement instruments, from a non-validated measurement between the apex of the lordotic curvature and a wooden ruler [11] to inclinometers [14] or even rastereography [16], and the selection of women at different stages of pregnancy, ranging from 17 to 34 weeks into pregnancy in some studies [15], make the comparison between studies difficult.

On the other hand, the activation of the extensor muscles during standing in healthy pregnant women may also change to face the increase in bending moment due to the enlarged abdominal mass. Nevertheless, there is no evidence about such adaptations during pregnancy, although they also may be related to the development of low back pain. In fact, the alterations of the activation patterns of the lumbopelvic extensor muscles have been related to low back pain in other populations [17,18].

In order to shed more light on postural changes and the extensor muscle activation during healthy pregnancy and postpartum, the aim of this study was to compare lumbar spine and pelvic curvatures, and muscle activation of the erector spinae and hamstring muscles between a group of pain-free nulliparous women, a group of pain-free pregnant women in the third trimester of pregnancy, and the same pregnant women postpartum, during erect standing. The hypothesis was that the biomechanical and morphological changes caused by pregnancy modify the position of the lower back and pelvis, as well as the activation of the extensor muscles.

## Methods

### Study design and setting

Data of this case control study were collected between September 2013 and June 2015 in a Biomechanics Laboratory.

### Participants

68 women participated in the study: 34 primiparous and multiparous in the third trimester of pregnancy, when morphometric changes are more evident (age: 34.7 ± 3.1; weeks of gestation: 36 ± 1) and 34 nulliparous who formed the control group (age: 32.9 ± 4.9).

Exclusion criteria for both the control and pregnancy groups were: (1) past or present low back pain, intense enough to cause work absence for illness, (2) spinal pathology (fractures, scoliosis, spondylolisthesis, spondylolysis, neoplastic processes, infections, vascular, metabolic or endocrine), (3) significant lower limb-length discrepancy and / or (4) past low back surgery. Additional exclusion criteria for the pregnancy group was any risk of abortion or any complications related to pregnancy.

Patients were recruited according to geographic proximity to the biomechanics laboratory. They were enrolled by midwives of different clinics from the same city. Only pregnant women in their third trimester of pregnancy were recruited, given that at this stage of pregnancy biomechanical changes are at their most evident. This research was approved by the Corporate Ethics Committee for Clinical Research in Primary Care of the Valencian Community, and the Ethics Committee on Human Research of the University of Valencia (Valencia, Spain). All the procedures were conducted in accordance with the principles of the World Medical Association’s Declaration of Helsinki and all participants provided their written informed consent.

### Instrumentation

The angular displacement of the lumbar spine and pelvis in the sagittal plane was recorded using a Liberty 240/16 electromagnetic motion capture system (Polhemus Inc., Colchester, USA), with an accuracy of 0.15° and a resolution of 0.00040° for sensor angular orientation.

This apparatus uses a low frequency magnetic field generated by an electromagnetic source, which is placed in a plastic platform adjusted at hip level for each participant. Two sensors detect the magnetic pulses, with a sampling frequency of 240 Hz. The first sensor (L1) was attached to the skin overlying the spinous process of the first lumbar vertebra, and provided data on the angular position of the trunk as a whole (lumbar and pelvis). The second sensor (S1) was placed at the level of the first sacral vertebra, and provided data on the inclination of the sacrum at the coxofemoral joint (hip flexion) [19]. Subtracting the S1 Data from the L1 data gave the angular position of the lumbar spine in the sagittal plane [20].

The EMG activity was recorded by two EMG100C Biopac modules (Biopac Systems, Inc., Goleta, CA), using pre-gelled disposable silver-silver chloride (Ag/AgCl) surface disk electrodes (2 cm diameter). Prior to EMG electrode placement, the registration points of the activity of each muscle were located following the recommendations of the Surface Electromyography for Non-Invasive Assessment of Muscles (SENIAM) project [21]. The hamstrings (biceps femoris) electrodes were placed at the midpoint of the distance between the right ischial tuberosity and the fibular head. The erector spinae EMG signal was recorded at the third lumbar vertebra, with electrodes placed 3 cm to the right of the spinous process. A reference electrode was placed at the level of the sternal body. After carefully cleaning and lightly abrading the skin with an alcohol pad, two recording electrodes were attached on each registration point, parallel to the underlying muscle fibers, with a center-to-center distance of 2 cm. The raw EMG signal was band-pass filtered (cutoff frequencies: 10 Hz high pass, 500 Hz low pass) and amplified (input impedance greater than 100 MΩ, common mode rejection ratio of 110 dB at 60 Hz, overall gain of 1000). EMG signals were A/D converted at a sampling frequency of 1000 Hz with a 16-bit data acquisition system (model MP150; Biopac Systems Inc.). A synchronic signal generated at the beginning of each measurement simultaneously sent a “start recording” order to both the electromagnetic motion capture system and the EMG recorder.

### Measurements and data processing

Age, height, weight and body mass index of all participants were recorded, as well as waist circumference before and after delivery in the pregnancy group.

Women adopted a barefoot standing position, with their feet pelvis-width apart, knees straight, arms by their sides, and with their hands facing inwards. Participants were told to stare at a mark set at eye level on the opposite wall, in order to avoid artefacts on the EMG signal caused by changes in head position [14,22,23]. They were asked to keep a natural, static, erect standing position: once the participant had adopted such position stably, angular displacement and EMG activity data were recorded for 5 seconds. All measurements were carried out once on the control group and twice on the pregnancy group, the first one at the third trimester of pregnancy, and the second one at an average of 8 ± 3 weeks postpartum.

All tests were carried out at least two hours after the subjects had risen from bed in order to minimize diurnal variations in their spinal mechanics [24].

EMG data were rectified and smoothed by calculating their root mean square, with a time window of 0.02 seconds. They were then normalized as a percentage of that muscle’s peak activity during a submaximal voluntary contraction, which has been found to be more reliable than maximum voluntary contractions for trunk muscle EMG normalization [25]. Prior to the standing position recordings, all the subjects performed a Biering-Sørensen maneuver (an isometric trunk extension with the upper body suspended in the air in front of the edge of a Roman chair) to normalize the erector spinae and biceps femoris signal [26]. MATLAB 2010a (MathWorks Inc., Massachusetts USA) was used for all data analysis.

In order to describe the postural pattern and EMG activation of the erector spinae and biceps femoris while standing, the variables used were the average degrees of lumbar flexion and rotation of the pelvis in the sagittal plane relative to the vertical, and the corresponding average percentage of activation of the lumbar erector spinae and biceps femoris.

### Statistical analysis

An a priori analysis of effect and sample size was made for a desired power of 90%. Effect size was estimated by means of Cohen’s d, calculated from the results of published work which compared similar dependent variables (EMG activity of the trunk extensor muscles, lumbopelvic position in the sagittal plane) between pregnant and non-pregnant women [14,27]. Sample size was calculated using the G*Power 3 software [28]. The result was an estimated minimum sample size of 32 subjects per group.

Compliance with the assumption of normality was checked for each dependent variable and each study group by means of the Kolmogorov-Smirnov test. In order to check the homogeneity in age and height between the control and pregnancy groups, a Student t-test for independent samples was used, with an alpha level of 0.05. Differences between the control and pregnancy groups, both ante and postpartum, were also analysed by means of the Student t-test for independent samples. Differences between measurements in pregnant women before and after childbirth were analysed by means of the Student t-test for related samples. To avoid the inflation of type I error due to repetition of pairwise comparisons, a Bonferroni adjustment was applied to the level of significance of these three comparisons (control vs postpartum, control vs antepartum and antepartum vs postpartum). Thus the alpha level for these three comparisons was 0.05/3, that is, 0.016. SPSS version 18.0 for Windows (SPSS Inc, Chicago, IL, USA) was used for all analysis.

## Results

Table 1 shows age, weight, height, body mass index and waist circumference of all the study groups. No significant differences in age and height were found between women in the control and pregnancy groups. Weight and body mass index were significantly lower in the control and postpartum groups respect to the pregnancy group (p <0.01), and abdominal circumference was higher in the antepartum respect to the postpartum measurements (p <0.01), as expected due to pregnancy-related changes in mass and body composition.

**Table 1:** Descriptive characteristics of the participants.

	Control group	Antepartum measurements	Postpartum measurements
n	34	34	34
Age (years)	32.9 ± 4.9 (25–45)	34.7 ± 3.1 (27–41)	35.0 ± 3.1 (27–42)
Weight (kg)	59.4 ± 8.6†	70.0 ± 8.7*	62.1 ± 8.7
Height (cm)	163.8 ± 5.5	163.2 ± 6.7	163.2 ± 6.7
Body mass index (kg / m^2^)	22.1 ± 2.9†	26.2 ± 2.7*	24.8 ± 9.8

Values presented as mean ± SD and range. P<0.01

*antepartum-postpartum

†antepartum-control.

Neither the degrees of lumbar flexion nor the rotation of the pelvis showed statistically significant differences between nulliparous, antepartum and postpartum measurements (Table 2).

**Table 2:** Lumbopelvic curvatures in the standing position for the three study groups.

	Control group	Antepartum measurements	Postpartum measurements
Lumbar (degrees)	-32.8 ± 9.2	-31.7 ± 10.5	-33.9 ± 9.3
Pelvis (degrees)	22.3 ± 8.2	21.3 ± 8.3	22.7 ± 7.6

Average degrees of lumbar and pelvic flexion (mean ± SD)

As for the EMG variables, the average EMG activation during erect standing of both the erector spinae and biceps femoris increased significantly in the group of pregnant women compared to both the nulliparous group and the postpartum measurements. Such differences, however, were not observed between the control group and the postpartum measurements for any of the EMG variables (Table 3).

**Table 3:** EMG percentages of activation in the standing position for the three study groups.

	Control group	Antepartum measurements	Postpartum measurements
Erector spinae (% Submax EMG)	2.4 ± 2.0†	4.6 ± 3.1*	2.1 ± 2.0
Biceps femoris (% Submax EMG)	1.2 ± 1.5†	3.4 ± 4.1*	1.4 ± 1.9

Average EMG activity expressed as a percentage of a submaximal voluntary contraction (mean ± SD).

P<0.01

*antepartum-postpartum

†antepartum-control.

## Discussion

This study analysed the effects of pregnancy on the position of the lumbopelvic region and the function of the trunk extensor and hamstring muscles in the standing position. Another of its objectives was to investigate whether these effects are maintained two months after delivery, compared to the non-pregnant pattern obtained from a control sample of nulliparous women. The results showed no statistically significant differences in the position of the lumbar spine or the pelvis between the three groups of measurements. That is, pregnancy in the third trimester did not cause postural alterations in the lumbopelvic region, compared to non-pregnancy or postpartum. With regard to muscle activity, both the lumbar and hip extensors showed a significantly increased activation during standing in the pregnancy measurements, compared with control and postpartum.

In the light of our results, pregnancy does not seem to modify the spinal curvatures during standing. The results obtained on the lumbopelvic position during standing are consistent with previous studies [11,13,14,16] in which no differences were observed in the degrees of lumbar curvature of pregnant women compared to the control group. These studies also included a follow-up of changes in lumbar curvatures during pregnancy by performing serial measurements, from the first to the third trimester. None of them found differences in lordotic curvature. Out of these studies, Ostgaard et al. [11] included the largest number of participants (855 women). The authors found no differences in the degree of lumbar curvature between 12 weeks and 36 weeks of pregnancy. Regarding the position of the pelvis, Bullock et al. [8], Gilleard et al [14] and more recently Betsch et al. [16], also found no pregnancy-related changes.

Nevertheless, the literature on lumbar curvature changes in the standing position related to pregnancy does not show unanimous results. Bullock et al. [8] and Otman et al. [7] found a significant increase in lumbar curvature throughout the three trimesters of pregnancy. Later, Franklin et al. [6], reached the same conclusion on a sample of 12 women, noting also an increased anterior rotation of the pelvis from the first to the third trimester of pregnancy. On the other hand, Okanishi et al. [15], in a more recent research, obtained the opposite result: a decrease in lumbar curvature and a posterior inclination of the sacrum. Such heterogeneity of results among the different studies may be explained in part by the use of different measurement instruments, from the use of an inclinometer and subsequent calculation of the lumbar curvature using a mathematical formula [7,8], to the use of electrogoniometers [6], video photogrammetry [14], photographs [15], or systems based on surface topography and 3D modelling [16]. The validity and reliability of some of these measurement systems for evaluating lumbopelvic motion has not been demonstrated [29]. In our study we measured lumbar spine and hip position, which is recorded by an electromagnetic motion capture system. Similar methods of lumbar curvature assessment, with two sensors of angular displacement at the same vertebral levels, have been used previously on low back pain patients [19, 20], even with the same motion capture system [23]. The reliability, accuracy, and low error rates of this kind of electromagnetic motion capture systems for the analysis of spinal motion are well known [30,31].

On the other hand, the small sample sizes used in some studies (9–15 women) [6,14,15] and the inclusion in the same group of women in very different stages of pregnancy, such as the gestational period from 16 to 35 weeks used by Okanishi et al. [15] as inclusion criteria, may also justify the variability in the results. Similar to the findings of Gilleard et al., [14] we have also observed a relatively large variance for each postural angle, which may indicate that subjects have an individual postural response to the increased trunk mass, as reported by Bullock-Saxton [8] and Moore et al.[13].

Similar to the results of the present study, Gilleard et al. [14] neither observed postural changes in pregnant women. Nevertheless, they found a significant reduction of the lumbar curvature 8 weeks after delivery, compared to nulliparous women. This contrasts with our results, given that we have not found differences postpartum. The small sample size used in their study may explain such differences between our results and theirs. The results of Betsch et al. [16] are also in line with ours. This study did not find alterations of lumbar curvature, either during pregnancy or at postpartum. This latter finding, which differs from observed Gilleard et al. [14], is consistent with that obtained in our study. Thus, in light of our results, pregnancy does not appear to modify spinal curvatures during standing.

With regard to muscle activity, our results showed a significant increase in erector spinae and biceps femoris activation in the standing position at the third trimester of pregnancy. This may be related to the increase in abdominal circumference and mass in the third trimester of pregnancy. Most people show an activation of the erector spinae muscles during standing [32–34], a fact which seems to be related to the anterior location of the center of gravity respective to the lumbar spine [34,35]. EMG activity of the erector spinae in the standing position has been analysed while holding objects of different weight and size, located at different distances from the body, finding increases in EMG activations related to increases in bending moment arms [35]. Thus, in the same way, in pregnant women the posterior muscles may be acting as active stabilizers, both of the lumbar spine and the pelvis, against the increased mass of the anterior part of the trunk and the corresponding increase in bending moment. In fact, the increased posterior muscle activity may be enough to compensate such increased bending moment, preventing it from causing adaptive changes in pelvic or spinal curvatures. On the other hand, the lack of differences between postpartum muscle activity and muscle activity in nulliparous women may be due to the significant reduction of abdominal volume after childbirth, which returns the bending moments exerted on the lumbar spine and pelvis to pre-pregnancy values, thus normalizing the demands of activation on the extensor muscles.

The results of this study challenge the traditional belief that pregnancy leads to an increase in lumbar curvature. Such increase has been thought to be behind the high incidence of low back pain in pregnant women, and in fact back pain treatment regimens given to pregnant and postpartum women include exercise for the reduction of lumbar curvatures through exercise [6]. Our results show that muscle responses, and not lumbar curvatures, are altered by pregnancy. This may be important in the design of new exercise regimens that are given to pregnant women.

This study had some limitations. In order to achieve the calculated sample size, and given the difficulties in recruiting this kind of participants, our sample was heterogeneous in terms of the number of previous pregnancies. This limitation, however, is shared by several previous studies with lower sample sizes [14,15,16]. Our study did not include follow-up measurements throughout pregnancy, and was limited to the last trimester of pregnancy. Future studies should investigate whether the number of previous pregnancies or the presence of low back pain influence the position and muscle activity of the lumbopelvic region and carry out serial measurements from the beginning to the end of pregnancy, in order to identify progressive alterations in these parameters.

This study focused on pain-free pregnant women, in order to shed light about the biomechanical adaptations shown by the spinal structures in the face of the altered mechanical demands associated to pregnancy. Since the current study has found no alterations in lumbopelvic curvatures during pregnancy when compared to nulliparous and postpartum women, other possible factors that may contribute to the high prevalence of low back pain in this population should be considered in future studies, such as changes in dynamic muscle activation patterns, alterations in ligamento-muscular responses or modifications in the distensibility of elastic structures. Such future studies should include not only pain-free pregnant women but also pregnant women with low back pain, in order to check if pregnancy-related spinal biomechanical adaptations change in this population.

## Conclusions

In standing, pregnant women in the third trimester of pregnancy do not show alterations in the position of the lumbar spine and pelvis respective to postpartum and nulliparous women.

In standing, pregnant women in the third trimester of pregnancy show an increased EMG activation of the lumbar and pelvic extensor muscles, respective to postpartum and nulliparous women. These results show how the trunk extensor muscles develop adaptative responses to the increase in abdominal volume.

## Supporting information

S1 Data This file includes all motion and EMG activity data used in this study. (XLSX)

## References

[1] Bastiaanssen JM, de Bie RA, Bastiaenen CH, Essed GG, van den Brandt PA. A historical perspective on pregnancy-related low back and/or pelvic girdle pain. Eur J Obstet Gynecol Reprod Biol. 2005; 1; 120(1):3–14. doi: 10.1016/j.ejogrb.2004.11.021 15866079 10.1016/j.ejogrb.2004.11.021

[2] Wu WH, Meijer OG, Uegaki K, Mens JM, van Dieen JH, Wuisman PI, et al Pregnancy-related pelvic girdle pain (PPP), I: Terminology, clinical presentation, and prevalence. Eur Spine J. 2004; 13(7):575–89. 10.1007/s00586-003-0615-y PMC3476662 15338362

[3] Kovacs FM, Garcia E, Royuela A, Gonzalez L, Abraira V. The Spanish Back Pain Research,Network. Prevalence and factors associated with low back pain and pelvic girdle pain during pregnancy. A multicenter study conducted in the spanish national health service. Spine (Phila Pa 1976). 2012; 37(17), 1516–33. 22333958 10.1097/BRS.0b013e31824dcb74

[4] Jensen RK, Doucet S, Treitz T. Changes in segment mass and mass distribution during pregnancy. J Biomech. 1996; 29(2):251–6. 8849820 10.1016/0021-9290(95)00042-9

[5] Opala-Berdzik A, Błaszczyk JW, Bacik B, Cieślińska-Świder J, Świder D, Sobota G, et al Static Postural Stability in Women during and after Pregnancy. A prospective Longitudinal Study. Plos One. 2015; 8;10(6):e0124207 doi: 10.1371/journal.pone.0124207 26053046 10.1371/journal.pone.0124207 PMC4460040

[6] Franklin ME, Conner-Kerr T. An analysis of posture and back pain in the first and third trimesters of pregnancy. J Orthop Sports Phys Ther. 1998; 28(3):133–8. doi: 10.2519/jospt.1998.28.3.133 9742469 10.2519/jospt.1998.28.3.133

[7] Otman AS, Beksac MS, Bagoze O. The importance of 'lumbar lordosis measurement device' application during pregnancy, and post-partum isometric exercise. Eur J Obstet Gynecol Reprod Biol. 1989; 31(2):155–62. 2759322 10.1016/0028-2243(89)90176-7

[8] Bullock JE, Jull GA, Bullock MI. The relationship of low back pain to postural changes during pregnancy. Australian Journal of Physiotherapy. 1987;33(1):10–7. doi: 10.1016/S0004-9514(14)60580-8 25025456 10.1016/S0004-9514(14)60580-8

[9] Gleeson PB, Pauls JA. Obstetrical physical therapy. review of the literature. Phys Ther. 1988; 68(11):1699–702. 3054943 10.1093/ptj/68.11.1699

[10] Fries EC HF. The influence of pregnancy on the location of the center of gravity, postural stability, and body alignment. Am J Obstet Gynecol. 1943; 46(3), 374–80.

[11] Ostgaard HC, Andersson GB, Schultz AB, Miller JA. Influence of some biomechanical factors on low-back pain in pregnancy. Spine (Phila Pa 1976). 1993; 18(1):61–5. 8434326 10.1097/00007632-199301000-00010

[12] To WW, Wong MW. Factors associated with back pain symptoms in pregnancy and the persistence of pain 2 years after pregnancy. Acta Obstet Gynecol Scand. 2003; 82(12):1086–91. 14616251 10.1046/j.1600-0412.2003.00235.x

[13] Moore K, Dumas GA, Reid JG. Postural changes associated with pregnancy and their relationship with low-back pain. Clin Biomech (Bristol, Avon). 1990; 5(3):169–74. 10.1016/0268-0033(90)90020-7 23916220

[14] Gilleard WL, Crosbie J, Smith R. Static trunk posture in sitting and standing during pregnancy and early postpartum. Arch Phys Med Rehabil. 2002; 83(12):1739–44. doi: 10.1053/apmr.2002.36069 12474180 10.1053/apmr.2002.36069

[15] Okanishi N, Kito N, Akiyama M, Yamamoto M. Spinal curvature and characteristics of postural change in pregnant women. Acta Obstet Gynecol Scand. 2012; 91(7), 856–61. doi: 10.1111/j.1600-0412.2012.01400.x 22429046 10.1111/j.1600-0412.2012.01400.x

[16] Betsch M, Wehrle R, Dor L, Rapp W, Jungbluth P, Hakimi M, et al Spinal posture and pelvic position during pregnancy: A prospective rasterstereographic pilot study. Eur Spine J. 2014; 24(6):1282–88. doi: 10.1007/s00586-014-3521-6 25155835 10.1007/s00586-014-3521-6

[17] Kim MH, Yoo WG. Comparison of the hamstring muscle activity and flexion-relaxation ratio between asymptomatic persons and computer work-related low back pain sufferers. J Phys Ther Sci. 2013; 25(5):535–6. doi: 10.1589/jpts.25.535 24259796 10.1589/jpts.25.535 PMC3804990

[18] Sánchez-Zuriaga D, Lopez-Pascual J, Garrido-Jaen D, Garcia-Mas MA. A comparison of lumbopelvic motion patterns and erector spinae behavior between asymptomatic subjects and patients with recurrent low back pain during pain-free periods. J Manipulative Physiol Ther. 2015; 38(2):130–7. doi: 10.1016/j.jmpt.2014.11.002 25499193 10.1016/j.jmpt.2014.11.002

[19] Mayer TG, Tencer AF, Kristoferson S, Mooney V. Use of noninvasive techniques for quantification of spinal range-of-motion in normal subjects and chronic low-back dysfunction patients. Spine (Phila Pa 1976). 1984; 9(6):588–95. 6238424 10.1097/00007632-198409000-00009

[20] Neblett R, Mayer TG, Gatchel RJ, Keeley J, Proctor T, Anagnostis C. Quantifying the lumbar flexion-relaxation phenomenon: Theory, normative data, and clinical applications. Spine (Phila Pa 1976). 2003; 28(13):1435–46. 12838103 10.1097/01.BRS.0000067085.46840.5A

[21] Hermens HJ, Freriks B, Disselhorst-Klug C, Rau G. Development of recommendations for SEMG sensors and sensor placement procedures. J Electromyogr Kinesiol. 2000; 10(5):361–74. 11018445 10.1016/s1050-6411(00)00027-4

[22] Opala-Berdzik A, Bacik B, Cieslinska-Swider J, Plewa M, Gajewska M. The influence of pregnancy on the location of the center of gravity in standing position. J Hum Kinet. 2010; 26: 5–11.

[23] Sánchez-Zuriaga D, Artacho-Pérez C, Biviá-Roig G. Lumbopelvic flexibility modulates neuromuscular responses during trunk flexion-extension. J Electromyogr Kinesiol. 2016; 28:152–7. doi: 10.1016/j.jelekin.2016.04.007 27155332 10.1016/j.jelekin.2016.04.007

[24] Adams MA, Dolan P, Hutton WC, Porter RW. Diurnal changes in spinal mechanics and their clinical significance. J Bone Joint Surg Br. 1990; 72(2):266–70. 2138156 10.1302/0301-620X.72B2.2138156

[25] Dankaerts W, O'Sullivan PB, Burnett AF, Straker LM, Danneels LA. Reliability of EMG measurements for trunk muscles during maximal and sub-maximal voluntary isometric contractions in healthy controls and CLBP patients. J Electromyogr Kinesiol. 2004; 14(3):333–42. doi: 10.1016/j.jelekin.2003.07.001 15094147 10.1016/j.jelekin.2003.07.001

[26] Biering-Sorensen F. Physical measurements as risk indicators for low-back trouble over a one-year period. Spine (Phila Pa 1976). 1984 3;9(2):106–19. 6233709 10.1097/00007632-198403000-00002

[27] Sihvonen T, Huttunen M, Makkonen M, Airaksinen O. Functional changes in back muscle activity correlate with pain intensity and prediction of low back pain during pregnancy. Arch Phys Med Rehabil. 1998 10;79(10):1210–2. 9779673 10.1016/s0003-9993(98)90264-7

[28] Faul F, Erdfelder E, Lang AG, Buchner A. G*Power 3: A flexible statistical power analysis program for the social, behavioral, and biomedical sciences. Behav Res Methods. 2007; 39(2):175–91. 17695343 10.3758/bf03193146

[29] Walsh M, Breen AC. Reliability and validity of the Metrecom Skeletal Analysis System in the assessment of sagittal plane lumbar angles. Clin Biomech (Bristol, Avon). 1995; 10(4):222–223. 10.1016/0268-0033(95)91402-z 11415557

[30] Pearcy M.J, Hindle R.J. New method for the non-invasive three-dimensional measurement of human back movement. Clin. Biomech. 1989; 4, 73–79. 10.1016/0268-0033(89)90042-9 23915997

[31] Jordan K., Haywood K.L., Dziedzic K., Garratt A.M., Jones P.W., Ong B.N., et al Assessment of the 3-dimensional Fastrak measurement system in measuring range of motion in ankylosing spondylitis. J. Rheumatol. 2004; 31, 2207–2215. 15517634

[32] Floyd WF, Silver PHS. Function of erectores spinae in flexion of the trunk. Lancet. 1951; 1(6647):133–4. 14795792 10.1016/s0140-6736(51)91212-3

[33] Carlsoo S. The static muscle load in different work positions: An electromyographic study. 1961;4(3):193–211.

[34] Klausen K, Rasmussen B. On the location of the line of gravity in relation to L5 in standing. Acta Physiol Scand. 1968; 72(1):45–52. doi: 10.1111/j.1748-1716.1968.tb03824.x 5655761 10.1111/j.1748-1716.1968.tb03824.x

[35] Kippers V, Parker AW. Electromyographic studies of erectores spinae: Symmetrical postures and sagittal trunk motion. Aust J Physiother. 1985; 31(3):95–105. doi: 10.1016/S0004-9514(14)60627-9 25025749 10.1016/S0004-9514(14)60627-9

